# Natamycin-Loaded Soy Protein Isolate/Cellulose Nanofibril Edible Composite Film for Improving the Postharvest Quality of Blueberries

**DOI:** 10.3390/foods15193495

**Published:** 2026-09-29

**Authors:** Haoru Qi, Xiaohui Pang, Sirui Wang, Bowen Tian, Shanshan Xie, Hongfei Zhao, Bolin Zhang

**Affiliations:** College of Biological Sciences and Technology, Beijing Key Laboratory of Forest Food Processing and Safety, Hebei Province Key Laboratory of Sustainable Utilization and Development of Forest Food Resources, Beijing Forestry University, No. 35 Qinghua East Road Haidian District, Beijing 100083, China

**Keywords:** SPI–CNF–NA composite film, soy protein isolate, cellulose nanofibers, blueberry, antifungal action

## Abstract

This study aimed to prepare a free-standing composite film consisting of soy protein isolate (SPI), cellulose nanofibers (3.0% CNF) and natamycin (3.0% NA) to maintain the freshness of postharvest blueberries, which are prone to spoilage and deterioration as a result of fungal infections. The antifungal performance of SPI–CNF–NA composite films against *Aspergillus niger* was determined via plate-based assay; its inhibition zone diameter against *A. niger* CICC 40868 reached 35.22 mm. Adding optimal amounts of CNF and NA maintained the SPI–CNF–NA composite film’s desirable buffer performance against water vapor, oxygen and ultraviolet light. In the blueberry preservation experiment, compared to the control group, SPI–CNF–NA composite film treatment reduced the weight loss rate of blueberries by 28.30%, and increased the contents of soluble solids, titratable acid and anthocyanins by 10.78%, 14.20%, and 33.13%, respectively. Applying the fabricated SPI–CNF–NA composite film not only maintained the freshness and exterior quality of postharvest blueberries, but also favored the preservation of their internal nutrients during a 20-day storage period. In summary, the addition of CNF and NA to prepare the SPI–CNF–NA composite film shows potential to improve the quality of postharvest blueberries. This study provides a theoretical and technical basis for the development of high-performance, stable biopolymer composite materials.

## 1. Introduction

Blueberry (*Vaccinium* spp.), as one of the representative berries of the *Vaccinium* genus, has attracted significant attention in the functional food industry thanks to its high nutritional and health values, attracting steadily increasing demand [1]. Postharvest fungal infection has been one of the main causes of blueberry spoilage and loss. Jiang et al. (2023) [2] have shown that, among the postharvest pathogenic fungi isolated from blueberry fruits, *Aspergillus niger* has the strongest pathogenicity and is prone to cause black-mold disease in blueberries, accelerating the decomposition of blueberry tissues and causing rapid rot of blueberry fruits. The quick deterioration of blueberries not only reduces their commercial value and edibility but also causes substantial economic losses [3]. Thus, the search for effective antifungal strategies to extend the shelf life of blueberries has attracted increasing attention in the industry [4].

Biodegradable food films fabricated from bio-based materials have emerged as a promising technology, driven by the green transformation. They are mainly applied in two forms: edible coatings and free-standing biofilm packaging. The former forms in situ on food surfaces without pouches, while the latter builds sealed packages that modulate internal gas (MAP) [5]. This study focuses on the latter. Although chitosan, alginate, and soy protein-based edible films have been widely applied for blueberry preservation, conventional protein-based wrappings still suffer from unsatisfactory water resistance and mechanical performance [6]. Findings have also indicated that papers on active packaging for the postharvest preservation of fruits and vegetables account for only 0.78% of all scientific papers published on this topic, demonstrating that this research direction remains largely underexplored [7]. Soybean protein isolate (SPI), as a naturally derived material, can be used as a matrix to fabricate composite films. Hu et al. (2025) [8] incorporated natural oleosomes extract into SPI films; the SPI/oleosome films effectively maintained firmness and microbial safety, preserving the vitamin C of refrigerated strawberries more effectively. However, pristine SPI films often suffer from poor mechanical properties and inadequate water barrier performance, which limits their application [9]. Cellulose nanofibers (CNF), as an environmentally-friendly biomass nanomaterial derived from abundant agricultural resources, can act as an effective plasticizing and reinforcing agent in films [10]. Qazanfarzadeh et al. (2016) [11] reported that the addition of oat hull-derived CNF increased the tensile strength of SPI-based films from 2.19 to 4.25 MPa. Xiao et al. (2020) [12] found that pure SPI and CNF films did not show any antibacterial activity, whereas the three ZnO-containing films exerted clear inhibitory effects on two common foodborne pathogens. This suggests that SPI–CNF composite films may not effectively control the *A. niger* causing blueberry spoilage. Thus, incorporating antifungal agents into SPI–CNF films could be a promising solution to reduce *A. niger* infection in blueberries.

Natamycin (NA), as an approved food additive and recognized as safe by the US Food and Drug Administration (FDA), is an odorless, non-rancid polyene macrolide compound produced by *Streptomyces* fermentation [13]. It exhibits potent inhibitory activity against fungal microorganisms, including *A. niger*, by interacting with sterols in cell membrane and thus disrupting membrane fluidity [14]. A report from Chen et al. (2025) [15] confirmed the enhanced antifungal activity of NA nanoparticles in chitosan, improving the storage quality of sweet cherries. However, no research on the use of SPI–CNF–NA edible composite films to inhibit *A. niger* for blueberry preservation has been reported yet. Beyond the intrinsic safety of the individual components, SPI, CNF, and NA show good mutual compatibility. The amphiphilic SPI matrix provides a continuous phase for homogeneous dispersion of CNF, while NA can be stably incorporated into the SPI–CNF network without triggering obvious phase separation or functional antagonism [16]. Economically, SPI benefits from sufficient supply and low cost as a commodity raw material derived from soybeans. CNF has seen decreasing production costs, though high-energy fibrillation limits large-scale, low-cost manufacturing. NA requires only low effective dosage despite its relatively high unit price. Therefore, this composite film is a potential solution for high-value blueberry preservation [17].

Thus, we are working on the assumption that incorporating NA into SPI–CNF composite systems could give the resulting films desirable performance potential. The objectives of this study were to: (1) use SPI and CNF as basic matrix and NA as an antifungal component to prepare a SPI–CNF–NA composite film; (2) characterize the structural, physical, and chemical properties of the composite film; and (3) evaluate the potential of the prepared edible composite film for preserving the freshness and extended shelf life of blueberries thanks to its antifungal effects.

## 2. Materials and Methods

### 2.1. Materials

Soybean hull was collected from Heze, Shandong Province, China. SPI (purity ≥ 85%) was purchased from Beijing Aoboxing Biotechnology Co., Ltd. (Beijing, China). Glycerol was obtained from Tianjin Yongda Reagent Co., Ltd. (Tianjin, China). Lyophilized powder of *Aspergillus niger* CICC 40868 (referred to as *A. niger* CICC 40868) was purchased from China Industrial Microbial Strain Preservation Center (CICC). Fresh blueberries, harvested in November 2025 from Honghe, Yunnan Province, China, at commercial harvest maturity (75–80 days after full bloom), were placed in plastic storage boxes and rapidly refrigerated at 4 °C within 8 h prior to experimental treatment. All other chemicals were of analytical grade.

### 2.2. Preparation of CNF from Soybean Hull

Following the method described in a previous study [18], soybean hull was ground into powder and sieved through a 60-mesh sieve, then mixed with distilled water, soaked for 12 h, and centrifuged at 8000 rpm for 5 min to collect the sediment. The sediment was mixed with a 5.0% NaOH solution at a ratio of 1:10 (g/mL), treated in a water bath at 60 ± 1 °C for 90 min, and centrifuged at 8000 rpm for 5min. Subsequently, the sediment was mixed with a NaClO_2_ solution at a ratio of 1:10 (g/mL), incubated for 30 min, and centrifuged. The sediment was washed with distilled water to neutrality, oven-dried at 60 ± 1 °C to constant weight, and sieved through an 80-mesh sieve, with a particle size of less than 0.3175 mm. The deep eutectic solvent (DES) was prepared from choline chloride and oxalic acid dihydrate (at a 1:1 molar ratio), with 30.0% (*w*/*w*) deionized water. The mixture was heated at 80 ± 1 °C until the solution became homogeneous and transparent. Next, the DES was mixed with the extracted soybean hull cellulose at a ratio of 1:10 (g/mL). The reaction system was incubated at 68 °C for 1.5 h and then centrifuged at 8000 rpm for 10 min. The collected precipitate was dialyzed against deionized water until the dialysate was neutral and then dried at 105 ± 1 °C to constant weight to obtain CNF. The CNF was ultrasonicated at 80 W for 5 min, followed by homogenization for 15 min.

### 2.3. Characterization of CNF

#### 2.3.1. Measurement of Micromorphology of CNF

Powder samples (10.0 mg) of soybean hull, cellulose, and CNF were fixed on conductive adhesive and sputter-coated with metal. Their surface morphologies were then observed using a high-resolution cold-field emission scanning electron microscope (Regulus8100, Hitachi, Tokyo, Japan) at an accelerating voltage of 3 kV. In addition, 5 μL aliquots of the CNF suspension were placed on a 200-mesh carbon-coated copper grid and dried at room temperature for 24 h. The morphology of CNF was observed using transmission electron microscopy (HT7800, Hitachi, Tokyo, Japan) at an accelerating voltage of 100 kV.

#### 2.3.2. Chemical Functional Group Determination of CNF

The dried soybean hull, cellulose, and CNF were treated with KBr for pellet preparation, and their chemical functional groups were characterized using FTIR (Nicolet iS50, Thermo Fisher Scientific, Waltham, MA, USA). Spectra were acquired with 32 co-added scans at a resolution of 4 cm^−1^ over a spectral range of 400 to 4000 cm^−1^.

#### 2.3.3. Analysis of Crystalline Structure of CNF

The crystalline structures of the soybean hull, cellulose, and CNF were analyzed using an X-ray diffractometer (Bruker D8 ADVANCE, Bruker, Waltham, MA, USA). The voltage was 40 kV, the current was 10 mA, and scans were carried out within the 2θ range of 5 to 40°. The scanning speed was 2°/min. The crystallinity (X) of the samples was calculated using Formula (1).

(1) $$X\;(\%)=\frac{\left(I_{002}\;-\;I_{\mathrm{am}}\right)}{I_{002}}\times 100$$

where *I*_002_ represents the maximum diffraction peak intensity at 2θ = 22.5°, and *I_am_* stands for the minimum intensity at 2θ = 18.0°.

#### 2.3.4. Determination of Thermal Property of CNF

The thermal properties of the soybean hull, cellulose, and CNF were evaluated using thermogravimetric analysis (TG 209 F3 Tarsus, Netzsch, Selb, Germany). Approximately 5 mg of each sample was weighed and loaded into alumina crucibles. Experiments were conducted under a N_2_ atmosphere with a flow rate of 40 mL/min and a heating rate of 10 °C/min, over a temperature range of 35 to 650 °C.

### 2.4. Preparation and Characterization of SPI–CNF–NA Composite Film

#### 2.4.1. Preparation of SPI–CNF–NA Composite Film

SPI film was prepared referring to the method of Zhu et al. (2016) [19], with a slight modification. First, 2.5 g of SPI and 1.25 g of glycerol were mixed into deionized water to prepare the SPI solution. Next, the SPI solution, after being homogenized at 800 rpm at room temperature for 5 min, was stirred at 75 ± 1 °C for 45 min to induce protein unfolding to expose the internal hydrophobic domains of SPI and facilitate intermolecular interactions for composite film fabrication. The unfolded SPI solution containing 20.0 mL of solution was poured into 15 cm Petri dishes and dried at 60 ± 1 °C to form the SPI film.

The optimal amounts of CNF and NA in the SPI–CNF–NA composite film were determined based on the mechanical properties, water vapor permeability, oxygen barrier property, and antifungal activity of the film. According to a previous method [20], first, CNF concentrations of 0%, 1.0%, 2.0%, 3.0%, 4.0%, and 5.0% (*w*/*w*) were mixed with 2.5 g of SPI and 1.25 g of glycerol dissolved in deionized water, and the final volume of the mixture was adjusted to 75 mL. As with the SPI film, the film-forming solutions containing SPI and CNF were homogenized at 800 rpm for 5 min at room temperature and then stirred at 75 ± 1 °C for 45 min to induce protein unfolding. A total of 20.0 mL of the solutions was poured into 15 cm Petri dishes and dried at 60 ± 1 °C to yield the SPI–CNF composite film. For the SPI–CNF–NA composite films, different concentrations of NA, ranging from 1.0 to 5.0%, were incorporated into SPI–CNF solution. As with the SPI–CNF films, the film-forming solutions containing SPI, CNF, and NA were homogenized at 800 rpm for 5 min at room temperature and then stirred at 75 ± 1 °C for 45 min to induce protein unfolding. Then, 20.0 mL of each solution was poured into a 15 cm Petri dishes and dried at 60 ± 1 °C to yield the SPI–CNF–NA composite films.

#### 2.4.2. Film-Property Analysis for Optimal Additive Content Selection

**Water vapor permeability of SPI–CNF and SPI–CNF–NA composite films**

Water vapor permeability was determined as previously described [21]. The SPI–CNF and SPI–CNF–NA films were placed over the openings of vials containing 3 g of anhydrous calcium chloride and sealed with elastic bands. The vials were placed in desiccators with deionized water at the bottom and stored at a constant temperature and humidity incubator at 25 °C. The weight of each vial was measured every 6 h. The water vapor transmission rate (WVTR) was calculated using Formula (2).

(2) $$\mathrm{WVTR}=\frac{M\;\times \;W}{\left(T\;\times \;S\;\times \;\Delta P\right)}$$

where *M* is the weight gain of the vial (g), *W* is the film thickness (m), *T* is the time (s), *S* is the permeation area (m^2^), and *ΔP* = 3167 Pa is the difference in water vapor partial pressure across the film.

**Oxygen permeability of the SPI–CNF and SPI–CNF–NA composite films**

Oxygen permeability (OP) was determined as previously described [22]. The SPI–CNF and SPI –CNF–NA composite films were placed over the opening of vials containing 3 g of deoxidizer (iron powder, activated carbon and sodium chloride at a mass ratio of 1:2:3) and sealed with elastic bands. The vials were placed in desiccators containing saturated barium chloride solution at the bottom and stored at a constant temperature and humidity incubator at 25 °C. The weight of each vial was measured every 6 h. OP was calculated using Formula (3).

(3) $$\mathrm{OP}=\frac{M\;\times \;W}{\left(T\;\times \;S\;\times \;\Delta P\right)}$$

where *M* is the weight gain of the vial (g), *W* is the film thickness (m), *T* is the time (s), *S* is the permeation area (m^2^), and *ΔP* = 21,278 Pa is the oxygen partial pressure difference across the film at 25 °C.

**Mechanical properties of SPI–CNF and SPI–CNF–NA composite films**

The tensile strength (TS) and elongation at break (EAB) of the SPI–CNF film were determined using an electronic universal testing machine (2025-01OST, Guangzhou Kunpeng Instruments Co., Ltd., Guangzhou, Guangdong, China). The SPI–CNF and SPI–CNF-NA composite films were cut into dumbbell shapes with a width of 1.0 cm and a length of 7.0 cm. The gauge length was 30 mm and the crosshead speed was 15 mm/min. Upon film rupture, the maximum tensile force and the membrane length between the clamps before and after stretching were recorded. The TS was calculated using Formula (4).

(4) $$\mathrm{TS}\;(\mathrm{MPa})=\frac{F_{\max}}{\left(L\;\times \;W\right)}$$

where *F_max_* is the maximum tensile force at rupture (N), *L* is the film thickness (mm), and *W* is the film width (mm).

The EAB was calculated using Formula (5).

(5) $$\mathrm{EAB}\;(\%)=\frac{\left(L_{2}\;-\;L_{1}\right)}{L_{1}}\times 100$$

where *L*_2_ is the film length at rupture (mm), and *L*_1_ is the initial film length (mm).

**Antifungal performance of SPI–CNF–NA composite film**

The antifungal activity of composite films against *A. niger* CICC 40,868 was evaluated using the agar well diffusion method according to the protocol reported by Dhadda et al. (2026) [23]. Briefly, activated strain spores were harvested and diluted to prepare a spore suspension at a concentration of 1.0 × 10^6^ CFU/mL. A total of 100 μL of the spore suspension was evenly spread onto a solidified potato dextrose agar (PDA) medium. After complete solidification, a sterile borer was used to punch a single well in the center of each agar plate. Subsequently, 100 μL of each film-forming solution containing SPI, CNF, and NA at concentrations ranging from 1.0 to 5.0% was loaded into individual wells. All the Petri dishes were incubated at 37 °C for 48 h. Then, the diameter of the transparent inhibition zone surrounding each well was measured using a digital vernier caliper to quantify the inhibitory efficacy against the growth of *A. niger* CICC 40868.

**Thickness of the three types of composite films**

The thickness of the three types of composite films was measured using a handheld micrometer (293-340, Mitutoyo Corporation, Kawasaki, Kanagawa, Japan).

**Microstructure observation of the three types of composite films**

The three types of prepared composite films were freeze-dried, fractured in liquid nitrogen to obtain intact cross-sections, and sputter-coated with a thin gold layer prior to observation using scanning electron microscopy (Regulus8100, Hitachi, Tokyo, Japan) at an accelerating voltage of 3 kV.

**Light barrier property of the three types of composite films**

The three types of composite films were cut into 1 cm × 3 cm rectangular pieces and placed tightly against one side of a quartz cuvette. The transmittance of the membranes was measured using a UV-visible spectrophotometer (UV-2550, Shimadzu Corporation, Kyoto, Japan) over a wavelength range of 200 to 800 nm to evaluate their light-blocking properties.

**Functional group analysis of the three types of composite films**

The chemical functional groups of the three types of composite films were analyzed using FTIR (Nicolet iS50, Thermo Fisher Scientific, MA, USA), with the attenuated total reflection method. Experiments were performed with 32 scans averaged, a resolution of 4 cm^−1^, and a spectral range of 400 to 4000 cm^−1^.

**Thermal properties of the three types of composite films**

The thermal stability of the three types of composite films was analyzed using a thermogravimetric analyzer (TG 209 F3 Tarsus, Netzsch, Selb, Germany). Approximately 5 mg of each sample was loaded into an alumina crucible and tested under a N_2_ atmosphere with a flow rate of 40 mL/min. The heating rate was 10 °C/min, and the temperature range was 35 to 650 °C.

### 2.5. Potential of the SPI–CNF–NA Composite Film for Preserving Blueberries

#### 2.5.1. Pretreatment of Blueberries

After being treated with distilled water and air-dried at room temperature for 20 min, the blueberries with no mechanical damage or obvious diseases were divided into four groups (100.0 g per group), following the procedure reported previously [24]. For the three treatment groups, the necks of conical flasks loaded with blueberries were individually covered with SPI, SPI–CNF and SPI–CNF–NA composite film, respectively, and fastened tightly using rubber bands. The fourth group, without any film wrapping, was designated as the control. All four groups of blueberries were stored in a refrigerator at 4 °C. Samples were collected on day 0, day 5, day 10, day 15, and day 20 of storage to determine the effects of the SPI–CNF–NA composite film on the freshness of the blueberries.

#### 2.5.2. Weight Loss Rate of Blueberry Fruits

The direct weighing method was adopted to observe the role of the SPI–CNF–NA composite film in reducing the loss of blueberries during the storage time. The weight of each group of blueberries was weighed and recorded every 5 days. It was calculated using Formula (6).

(6) $$\mathrm{Weight loss rate}\;(\%)=\frac{\left(m_{0}\;-\;m_{t}\right)}{m_{0}}\times 100$$

where *m*_0_ represents the initial weight of the blueberries (g) and *m_t_* represents the weight of the blueberries on the t-th day of storage (g).

#### 2.5.3. Decay Rate of Blueberries

The decay condition of blueberries is classified into four grades: Grade 0, with no obvious changes on the surface; Grade 1, with obvious softening phenomenon; Grade 2, with severe softening and leakage of juice; and Grade 3, with surface infection by mold spots [25]. The decay rate was calculated using Formula (7) to determine the potential of SPI–CNF–NA composite film for lowering the loss of blueberries.

(7) $$\mathrm{Decay rate}\;(\%)=\frac{\Sigma (G_{i}\;\times \;N_{i})}{\left(G_{\max}\;\times \;N_{0}\right)}\times 100$$

where *G_i_* denotes the decay level at the i-th stage, *N_i_* is the number of decayed fruits at the i-th stage, *G_max_* represents the maximum decay level, and *N*_0_ refers to the total number of blueberries.

#### 2.5.4. Appearance Performance and Firmness of Blueberries

Three blueberry fruits randomly selected from each test group were photographed to record the change in blueberry fruit appearance. The firmness of blueberries hardness was expressed in Newtons (N) using texture analyzer (TVT-6700, Perten Instruments, Huddinge, Sweden). The test speed was 0.5 mm/s, the trigger force was 5.0 g, the penetration depth was 50.0% of the blueberry diameter.

#### 2.5.5. The Total Soluble Solid Content (TSS) of Blueberries

The TSS (expressed as °Brix) of blueberries was determined using a handheld refractometer (HZ-80B, Guangzhou Huazhi Instruments Co., Ltd., Guangzhou, Guangdong, China). For each treatment group, ten blueberries were randomly selected, fully ground and filtered through four layers of cheesecloth. The filtrate was then dropped onto the prism surface of the refractometer. The reading was observed and recorded.

#### 2.5.6. The Total Titratable Acidity (TTA) of Blueberries

The blueberries were ground evenly in a mortar, and 1.0 g of the ground pulp was mixed with 20.0 mL of distilled water in a conical flask. The mixture was then heated in a water bath of 80 ± 1 °C for 0.5 h and then cooled to room temperature. Phenolphthalein indicator (2–3 drops) was added to the mixture, followed by titration with 0.1 mol/L NaOH solution using a burette until the mixture turned pink, with the color persisting for 30 s. The burette readings for the NaOH solution before and after titration were recorded. The total titratable acidity (TTA) of blueberries was calculated using Formula (8).

(8) $$\mathrm{TTA}\;(\%)=\frac{c\times \;(V1-V2)\;\times \;k\;\times \;F\;\times \;100}{m}$$

where *c* represents the concentration of the NaOH (mol/L); *V*_1_ represents the volume of the NaOH consumed during the titration (mL); *V*_2_ represents the volume of the NaOH consumed in the control experiment (mL); *k* represents the conversion factor for acid—for berries, citric acid containing one molecule of crystal water was used as the reference acid, with a corresponding conversion factor of 0.070; *F* represents the dilution factor; and m represents the mass of the blueberries (g).

#### 2.5.7. The Anthocyanin Content of Blueberries

The anthocyanin content (TAC) of treated blueberries was determined as described by Xu et al. (2023) [26]. Mortar-ground blueberry homogenate (1.0 g) was mixed with 25.0 mL of 80% ethanol, followed by dark extraction at room temperature for 2 h, and centrifuged to collect the filtrate. A 1.0 mL aliquot of the filtrate was diluted to 5.0 mL with a pH 1.0 potassium chloride buffer and pH 5.0 sodium citrate buffer separately, using deionized water as the control. Absorbance was measured at 510 nm using a UV-visible spectrophotometer (T6, Beijing Persee General Instruments Co., Ltd., Beijing, China). The TAC of blueberries was calculated according to Formula (9).

(9) $$\mathrm{TAC}\;(\mathrm{mg}/g)=\frac{\Delta A\times \;\mathrm{Mw}\;\times \;\mathrm{Df}\;\times \;V\;\times \;L}{\left(\varepsilon \;\times \;m\right)}$$

where *ΔA* is the absorbance difference at 510 nm between the pH 1.0 and pH 5.0 solutions; *Mw* = 449.2 g/mol (molecular weight of cyanidin-3-O-glucoside); *D_f_* denotes the dilution factor; *V* represents the total volume of the extract (mL); *L* = 1.0 cm; ε = 29,600 L/(mol·cm) (molar absorptivity of cyanidin-3-O-glucoside); and m is the weight of the blueberries (g).

#### 2.5.8. Microbiological Numeration of Blueberries

According to the Wang et al. (2023) [27], 25.0 g of treated blueberries was ground under sterile conditions and suspended in 225.0 mL of sterile water, before being vortexed for 2 min to uniformly disperse surface-associated bacteria, yeasts, and molds. The supernatant was serially diluted, and 1.0 mL of the homogenate from each dilution gradient was accurately pipetted into Petri dishes which were incubated at 37 °C for 48 h to determine the live counts of bacteria, yeast, and mold according to Chinese National Standard GB 4789.2-2022 [28]. A Luri–Bertani (LB) medium was used to enumerate total bacteria, and PDA medium was used to count yeasts and molds.

### 2.6. Statistical Analysis

All experiments were independently performed in triplicates. Statistical analyses were conducted using SPSS Statistics 27; Image J software (version 1.54p) was used for image-based quantitative measurements; and one-way analysis of variance (ANOVA) was employed to assess statistical significance. A *p*-value < 0.05 is considered statistically significant. All data are presented as the mean ± standard deviation, and graphs are generated with the Origin 2024.

## 3. Results

### 3.1. Characterizations and Preparation of CNF

#### 3.1.1. The Appearance and Micromorphology of CNF

**Figure 1:**
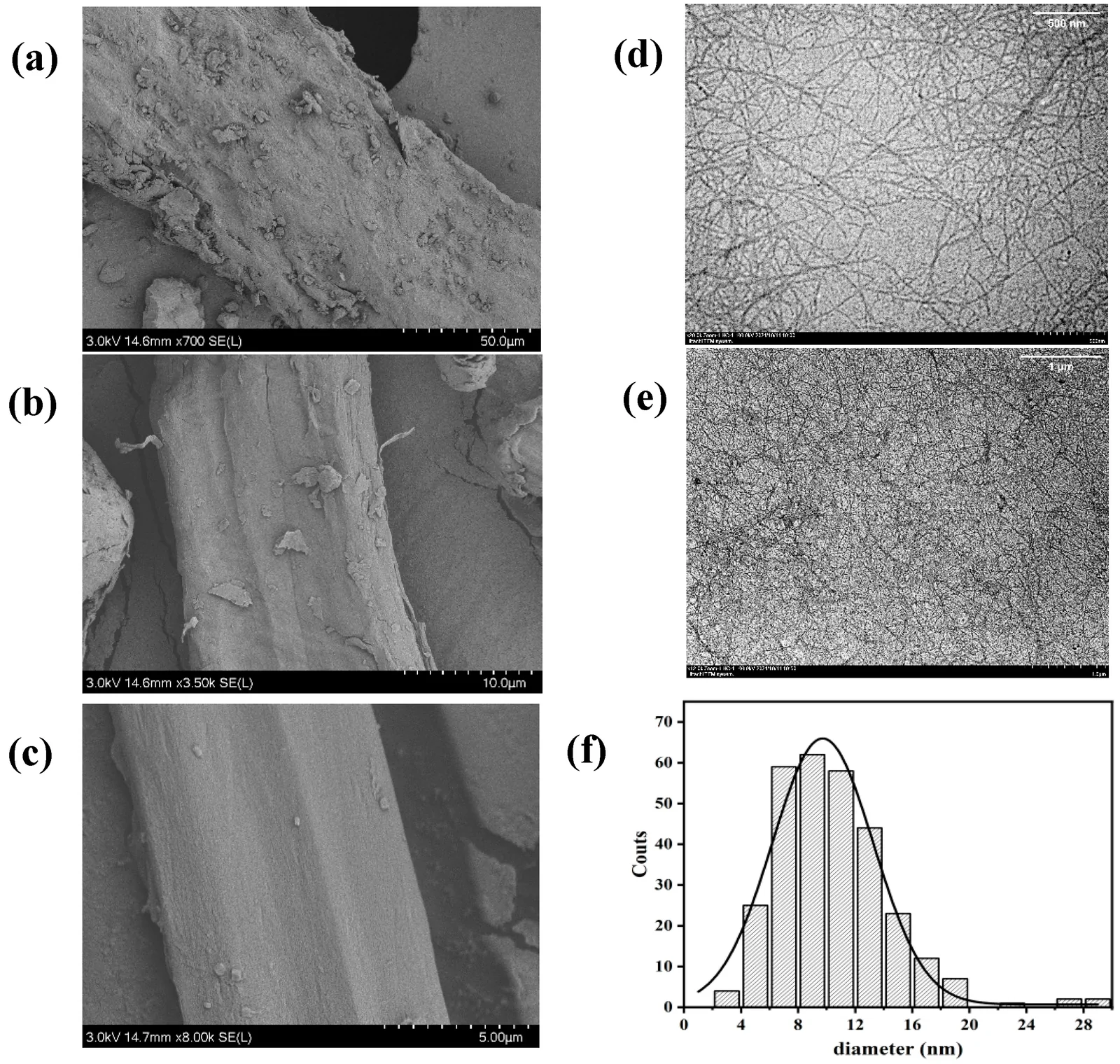
Micromorphological characterization of soybean hull, cellulose, and CNF. (**a**) SEM image of soybean hull powder; (**b**) SEM image of cellulose; (**c**) SEM image of CNF; (**d**,**e**) TEM images of CNF at 12,000× (**d**) and 20,000× (**e**) magnifications, showing rod-like nanoparticles; (**f**) particle size distribution of CNF derived from TEM images.

As shown in Figure 1a–c, soybean hull exhibited an uneven and heterogeneous structure, with a notably rough appearance. After alkaline treatment and bleaching to remove impurities, the cellulose was effectively separated, leading to cellulose and CNF with relatively smoother surfaces. Moreover, TEM images with magnifications of CNF 12 k and 20 k Figure 1d,e) show that CNF has a long rod-like or filamentous structure without obvious aggregation or fragmentation. The widths of 300 individual CNFs were measured from representative TEM images using Image J software. Gaussian fitting was performed, and the fitted diameter of CNF was approximately 9.71 nm, as shown in Figure 1f.

#### 3.1.2. Chemical Functional Group of CNF

**Figure 2:**
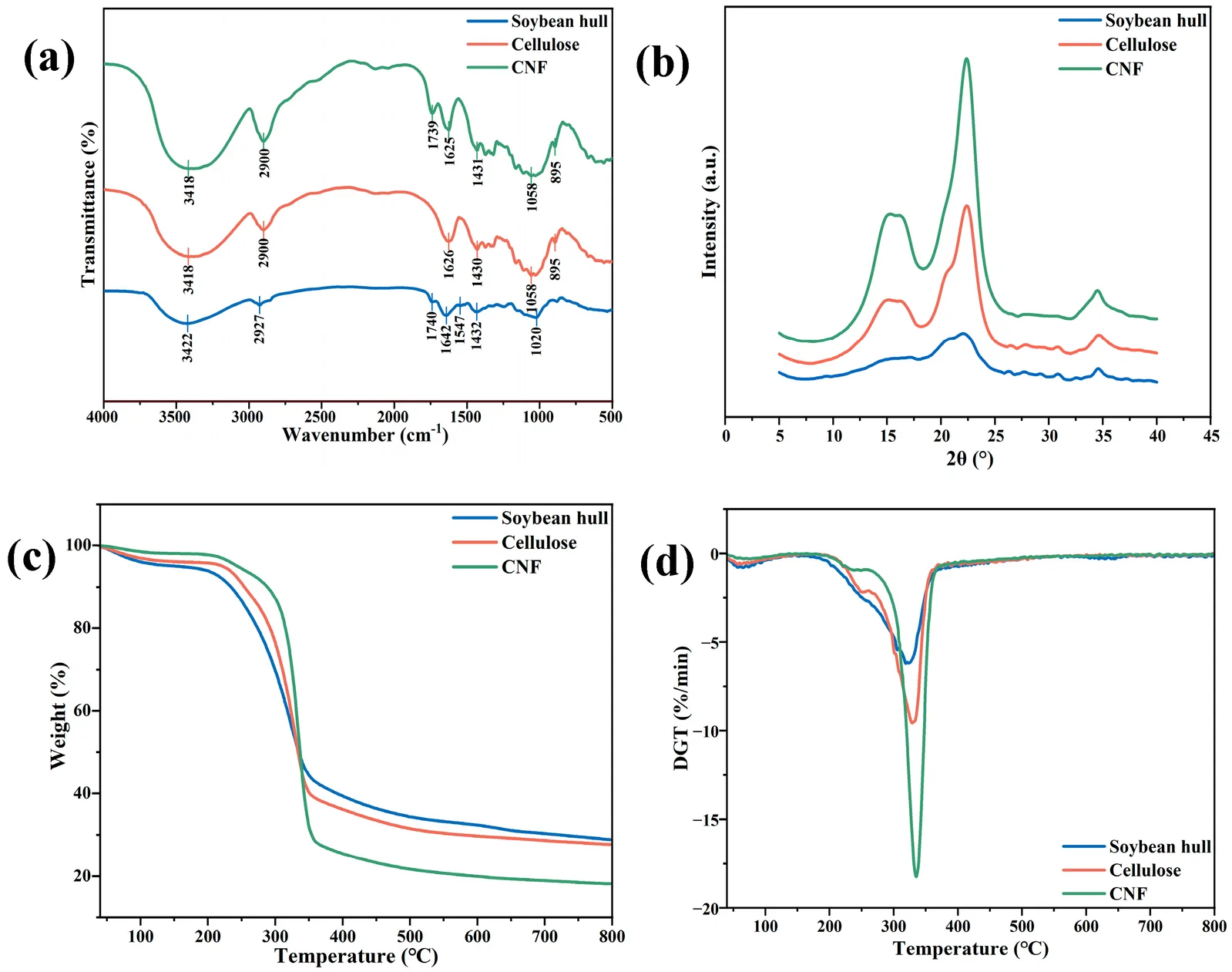
Spectroscopic and thermal characterization of soybean hull, cellulose, and CNF. (**a**) FTIR spectra of soybean hull, cellulose, and CNF; (**b**) XRD patterns of soybean hull, cellulose, and CNF; (**c**) TGA curves of soybean hull, cellulose, and CNF; (**d**) DTG curves of soybean hulls, cellulose, and CNF.

The chemical functional groups of soybean hull, cellulose, and CNF were characterized using FTIR. As shown in Figure 2a, soybean hulls exhibit absorption bands at 895, 1020, 1430, 1640, 2900, and 3420 cm^−1^. The signals at 1740 and 1547 cm^−1^ are characteristic bands of soybean hulls, which are completely absent in purified cellulose samples. Compared with cellulose, CNF displayed an additional characteristic peak at 1739 cm^−1^. Furthermore, CNF shows significantly higher peak intensities than both soybean hull and cellulose, with the 3418 cm^−1^ O–H stretching peak exhibiting the most pronounced enhancement.

#### 3.1.3. The Crystalline Structure of CNF

Hemicellulose and lignin are inherently amorphous in nature, while cellulose consists of both crystalline and amorphous domains. Cellulose polymorphs are primarily classified into four types, with Type I and Type II being the predominant forms characterized by XRD. As depicted in Figure 2b, soybean hull, cellulose and CNF exhibited characteristic diffraction peaks at 15.2°, 22.3° and 34.5°, respectively. Quantitative XRD analysis revealed that the crystallinities of cellulose and CNF were 62% and 67%, respectively.

#### 3.1.4. The Thermal Property of CNF

The TGA and DGT results are presented in Figure 2c,d. Soybean hull, cellulose and CNF exhibited three distinct mass loss stages across the tested temperature range. First, the recorded mass losses were 4.93% for soybean hull, 4.89% for cellulose, and 1.92% for CNF during a low-temperature stage of 50 to 150 °C. Second, the recorded mass losses were 50.44% for soybean hull, 54.59% for cellulose, and 65.32% for CNF during the primary thermal degradation stage of 200 to 350 °C. Third, residual oxidative decomposition occurred during the third stage, between 350 and 650 °C. Meanwhile, the onset thermal decomposition temperatures were calculated to be 319 °C for soybean hull, 328 °C for cellulose, and 335 °C for CNF. Clearly, CNF had a lower residual mass than soybean hull and cellulose, indicating that CNF exhibits superior thermal stability.

Overall, our results confirmed that highly pure nanoscale cellulose, i.e., CNF, was successfully produced by removing impurities such as hemicellulose and lignin from soybean hulls through alkaline and bleaching treatments, followed by DES treatment for cellulose. The prepared CNF has a higher thermal stability than soybean hull and cellulose.

### 3.2. Preparation and Characterization of the SPI–CNF–NA Composite Film

#### 3.2.1. Preparation of SPI–CNF–NA Composite Film

**Figure 3:**
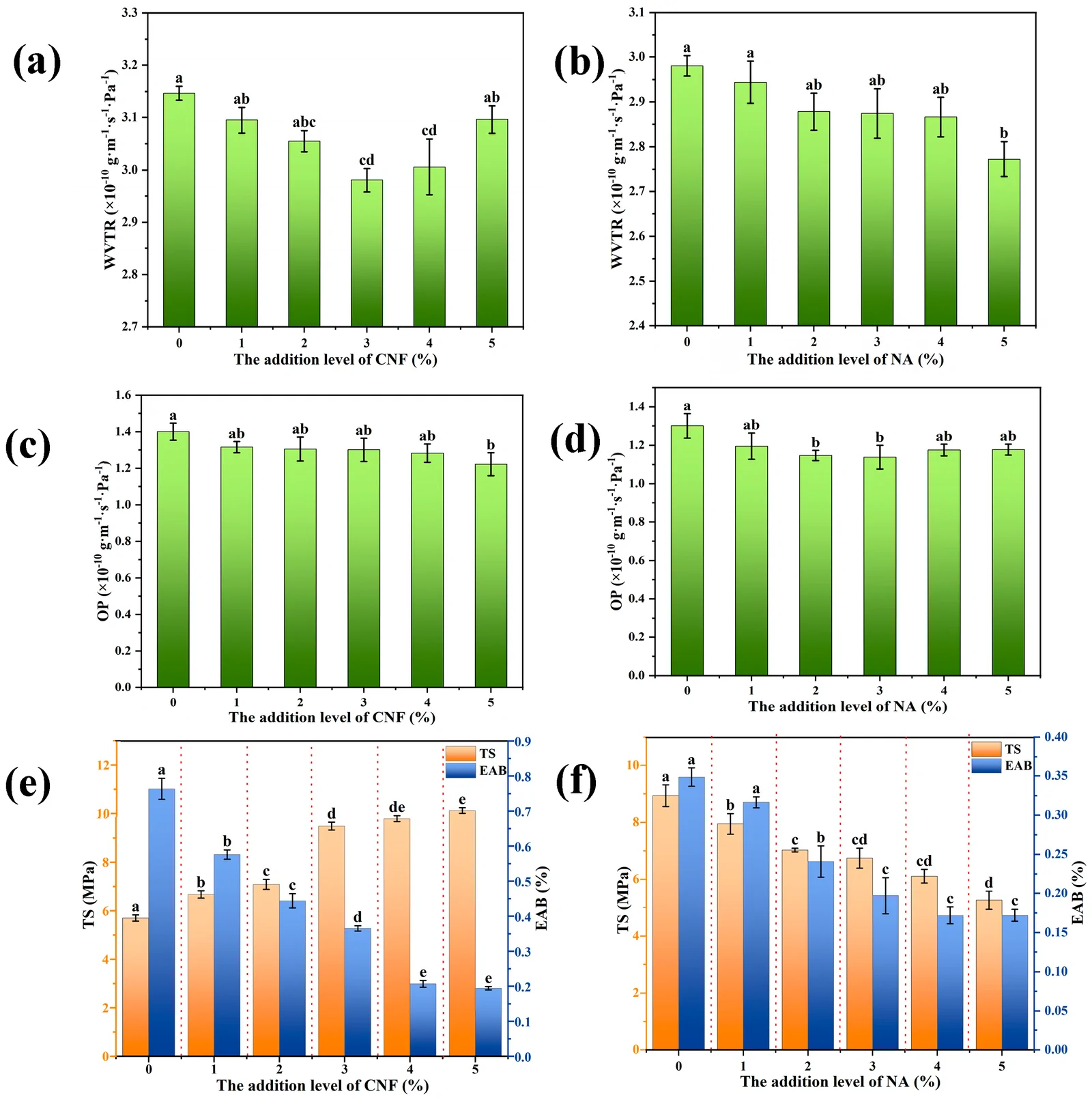
Effects of CNF and NA loadings on the physicochemical and mechanical properties of SPI–CNF and SPI–CNF–NA composite films. (**a**) WVTR as a function of CNF loading; (**b**) WVTR as a function of NA loading; (**c**) OP as a function of CNF loading; (**d**) OP as a function of NA loading; (**e**,**f**) TS and EAB of films as a function of CNF (**e**) and NA (**f**) loadings, respectively. Data are expressed as mean ± SD (n = 3). Different lowercase letters denote significant differences among groups at *p* < 0.05, as determined by one-way ANOVA followed by Tukey’s multiple range test.

**Water vapor transmission rate of SPI–CNF and SPI–CNF–NA composite films.** As depicted in Figure 3a,b, the amounts of CNF and NA added directly affected water vapor migration in the two composite films from the internal to the external ambient atmosphere. For the SPI–CNF composite film, the WVTR first decreased and then increased as the CNF level increased. A minimum of 2.98 × 10^−10^ g/(m·s·Pa) of WVTR was observed when 3.0% CNF was added to SPI–CNF composite film. It was seen that, when the amount of CNF added was higher than 3.0%, the WVTR values of the SPI–CNF composite film gradually increased, but they were lower than that of the control group. Regarding the SPI–CNF–NA composite film, the WVTR values gradually decreased from 2.98 × 10^−10^ to 2.77 × 10^−10^ g/(m·s·Pa) as the amount of NA added increased from 1.0 to 5.0%. It is interesting to note that when the amount of NA added was 3.0%, the WVTR value of the SPI–CNF–NA composite film was 2.87 × 10^−10^ g/(m·s·Pa), which is a satisfactory value for effectively controlling the water vapor permeability of SPI–CNF–NA composite film.

**Oxygen permeability of SPI–CNF and SPI–CNF–NA composite films.** As illustrated in Figure 3c,d, the OP values showed a decreasing trend as the CNF content increased. When the amount of CNF added increased from 1.0 to 3.0%, the OP values of the SPI–CNF composite film decreased from 1.40 × 10^−10^ to 1.30 × 10^−10^ g/(m·s·Pa). It was seen that when 1.0% NA was added to the SPI–CNF composite film, the OP value of the SPI–CNF–NA composite film directly decreased to 1.19 × 10^−10^ g/(m·s·Pa). When the amount of NA added was 3.0%, the SPI–CNF–NA composite film had the lowest OP value (Figure 3d). In summary, the incorporation of CNF and NA effectively improved the oxygen barrier property of SPI-based composite films.

**Mechanical properties of SPI–CNF and SPI–CNF–NA composite films.** The mechanical properties of two composite films, after the addition of CNF and NA, are displayed in Figure 3e,f. Regarding the SPI–CNF composite film, increasing the CNF level caused its TS value to undergo a progressive upward trend. The TS value of the SPI–CNF composite film rose from 7.10 to 8.93 MPa when the CNF level increased from 2.0 to 3.0%. It was noted that the TS increment of the SPI–CNF composite film would slow down if the amount of CNF added was higher than 3.0%. At the same time, the EAB value of the SPI–CNF composite film showed a marked reduction from 34.84 to 21.20% if the CNF content was increased from 3.0 to 4.0%. Moreover, adding NA significantly decreased the TS and EAB values of the SPI–CNF–NA composite film. When the amount of NA added was 3.0%, the TS and EAB values of the SPI–CNF–NA composite film were reduced to 6.74 MPa and 19.70%, respectively. Clearly, selecting an adequate adding level of NA is important for maintaining the mechanical properties of SPI–CNF–NA composite film with balanced TS and EBA values.

**Figure 4:**
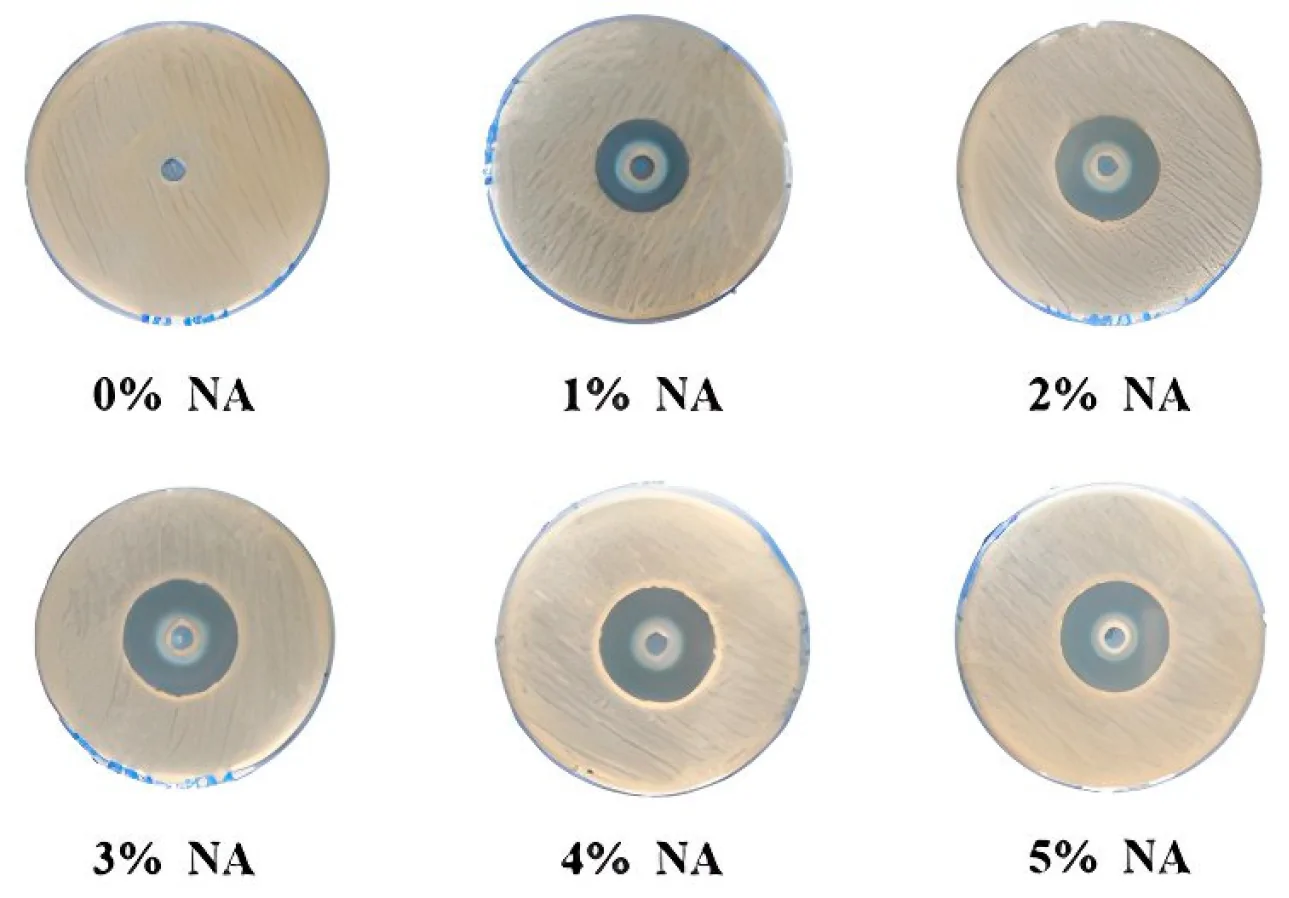
Antimicrobial activity of NA against tested strain *A. niger* CICC 40,868 by inhibition zone.

**The antifungal property of SPI–CNF–NA composite film.** The effect of the amount of NA added on the inhibitory ability of the SPI–CNF–NA composite film against *A. niger* is depicted in Figure 4 and summarized in Table 1. It was seen that the NA-free SPI–CNF film solution did not exhibit any antifungal activity against the growth of *A. niger*, confirming the lack of inherent antifungal properties of SPI or CNF. When the amount of NA added ranged from 1.0 to 3.0%, the inhibition zone diameter (IZD) of the SPI–CNF–NA composite film solution against *A. niger* increased progressively from 27.52 to 34.09 mm. It was noted that the IZD increased by only 1.13 mm, although the addition level of NA increased from 3.0 to 4.0%. Clearly, adding more NA did not significantly promote the capacity of the composite film to inhibit the growth of *A. niger*.

In summary, our results showed that the optimal CNF and NA-loading levels are 3.0% for the preparation of SPI–CNF–NA composite film. In this case, the involvement of CNF and NA not only improved the overall performance of the SPI-based film in terms of water vapor transmission, oxygen permeability and thermal stability but also, importantly, gave it good antifungal capacity.

#### 3.2.2. Characterization of SPI, SPI–CNF and SPI–CNF–NA Composite Films

**Figure 5:**
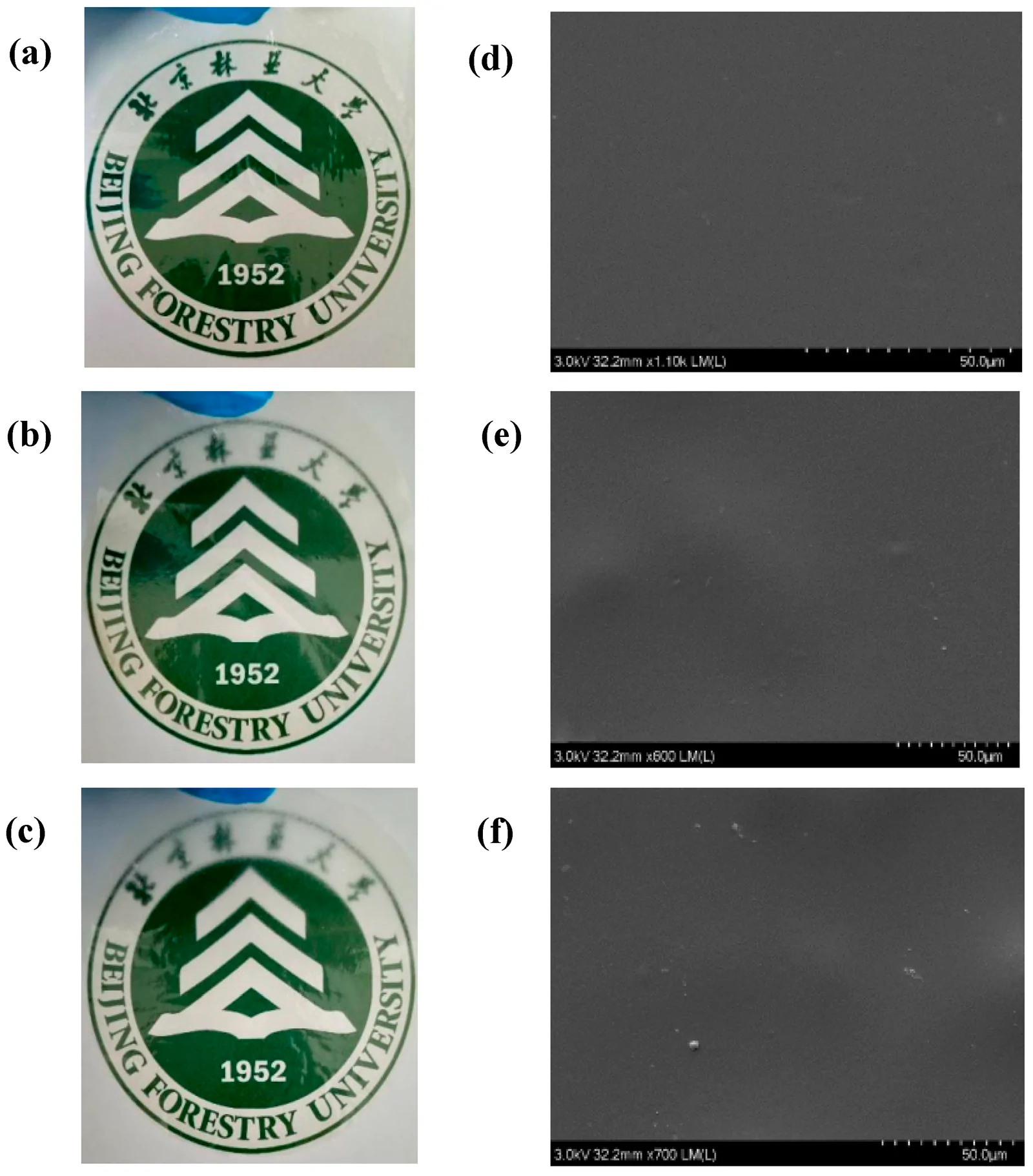
Optical transparency and surface morphology of the SPI, SPI–CNF, and SPI–CNF–NA composite films. (**a**–**c**) Photographs of the SPI (**a**), SPI–CNF (**b**), and SPI–CNF–NA (**c**) films placed over the school emblem; (**d**–**f**) SEM images showing the surface morphologies of the SPI (**d**), SPI–CNF (**e**), and SPI–CNF–NA (**f**) films.

**Transparency and SEM of the three films.** The effect of the addition of CNF and NA on the appearance properties of SPI–CNF and SPI–CNF–NA composite films is depicted in Figure 5a–c. The incorporation of CNF (3.0%) and NA (3.0%) enhanced the opacity of the two SPI-based films. Interestingly, the school emblem of our institution (Beijing Forestry University) remained clearly visible when covered by the SPI–CNF and SPI–CNF–NA composite films, confirming the opacity of the two SPI-based films due to the presence of CNF and NA. SEM observation showed that no obvious large particles were visible on the surface of the two films (Figure 5d–f). However, the SPI–CNF–NA composite film had a slightly rough surface, with dispersed small particles, compared to the smooth, homogeneous surfaces of the SPI and SPI–CNF films.

**Figure 6:**
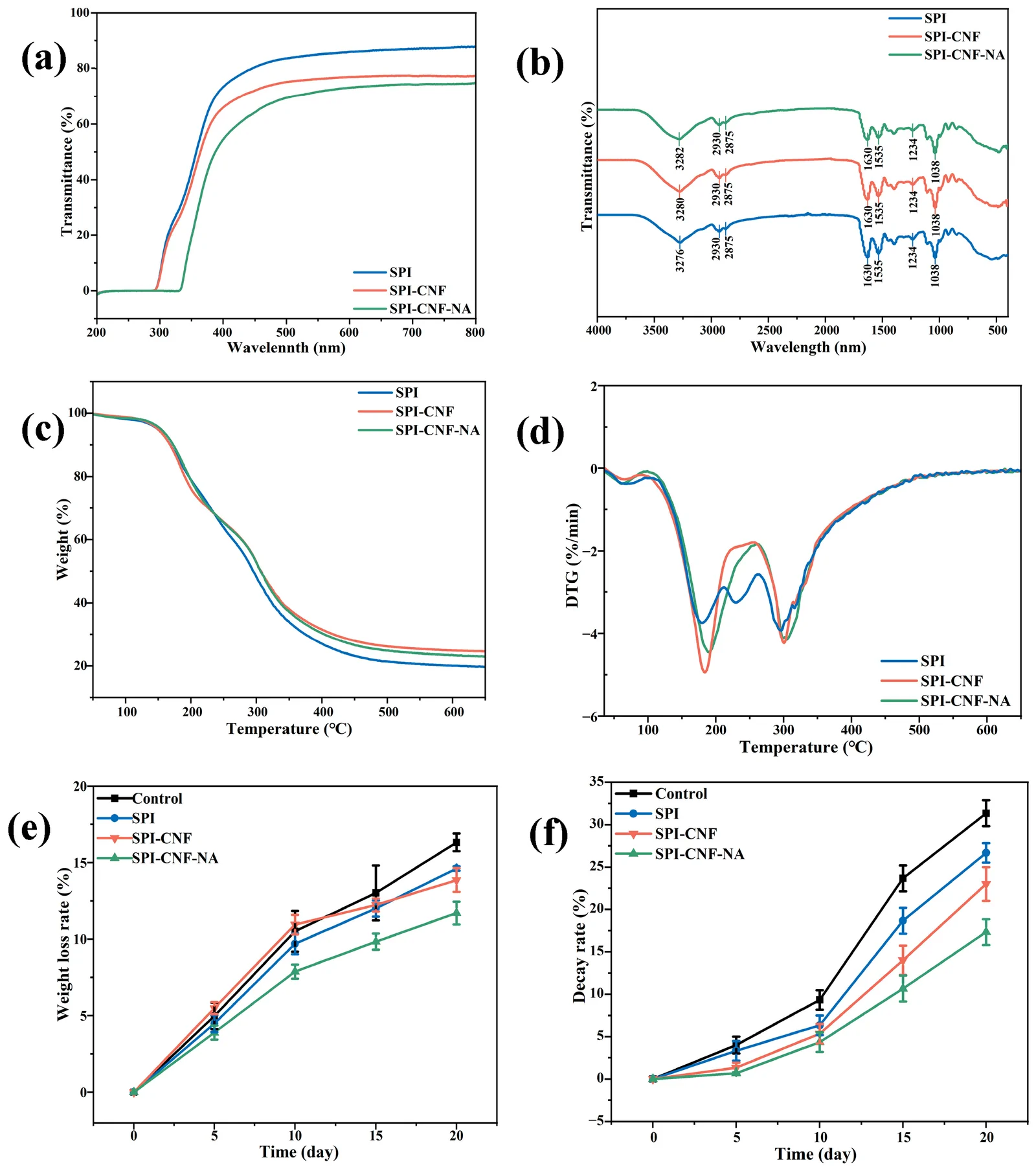
Physicochemical properties of three SPI-based composite films and their effects on the preservation of blueberries during a 20-day storage period. (**a**) Light transmittance of the SPI, SPI–CNF, and SPI–CNF–NA composite films; (**b**) FTIR spectra of the SPI, SPI–CNF, and SPI–CNF–NA composite films, (**c**) TGA curves of the SPI, SPI–CNF, and SPI–CNF–NA composite films, and (**d**) DTG curves of the SPI, SPI–CNF, and SPI–CNF–NA composite films; (**e**) weight loss rate of treated blueberries, (**f**) decay incidence of treated blueberries.

**Light barrier properties of the three films.** The light transmission rate of the three films is presented in Figure 6a. It indicates that the light transmittance rate of the SPI, SPI–CNF and SPI–CNF–NA composite films in the visible light region were 87.74%, 77.30% and 74.79%, respectively. The light transmittance rates of the three SPI-based films in the UV light region were 73.31%, 65.91% and 54.41%, respectively. Clearly, the addition of CNF and NA decreased the light transmittance rate of the SPI–CNF and SPI–CNF–NA composite films compared to the SPI film. Moreover, a remarkably low UV light transmittance rate was seen for the three SPI-based films in wavelengths ranging from 200 to 300 nm (Figure 6a). This suggests that the addition of CNF and NA allowed the prepared SPI-based films to perform better as transparent materials with effective UV barrier properties.

**FTIR description of the three films.** The evolution of the molecular vibration modes of the three SPI-based films, reflecting the interactions among their different components, was depicted using the FTIR technique (Figure 6b). It can be seen that the SPI film has broad absorption peaks ranging from 3200 to 3650 cm^−1^ as well as a peak at 3060 to 3330 cm^−1^, corresponding to O–H and N–H stretching vibrations. The peaks at 2930 and 2875 cm^−1^ are attributed to C–H stretching vibrations. The characteristic peaks at 1630 cm^−1^, 1535 cm^−1^, and 1234 cm^−1^ are assigned to the amide I (C=O stretching), amide II (N-H bending), and amide III (C-N stretching) bands of the SPI film, respectively. Compared to the FTIR spectra of the SPI film, no new absorption peaks are observed in the SPI–CNF or SPI–CNF–NA composite films. However, the addition of CNF and NA lead to shifts in the absorption peak from 3276 to 3280 cm^−1^ for the SPI–CNF film and to 3282 cm^−1^ for the SPI–CNF–NA composite film. As a result, the absorption intensities of the amide I, II, and III bands in the SPI–CNF and SPI–CNF–NA composite films are lower than those in the SPI film. Notably, the peak at 1038 cm^−1^, corresponding to the C–O stretching vibration of glycerol, is sharper for the SPI–CNF film but is attenuated for the SPI–CNF–NA composite film.

**Thermogravimetric analysis of the three films.** The effect of the addition of CNF and NA on the tolerance of SPI, SPI–CNF and SPI–CNF–NA composite films to thermal treatment is displayed in Figure 6c,d. The SPI film exhibited a three-stage thermal decomposition process, as below, with different substances lost at each stage. The second stage represents the primary thermal degradation step. However, the SPI–CNF and SPI–CNF–NA composite films only underwent a two-stage thermal decomposition process. The thermal weight loss of the three SPI-based films, after being treated under temperatures ranging from 240 to 260 °C, was ranked as SPI > SPI–CNF–NA > SPI–CNF.

Overall, the results of our study support the fact that combining SPI, as a basic material, with CNF and NA holds potential for the preparation of SPI–CNF–NA composite film. In addition to good mechanical properties, including water vapor permeability, oxygen permeability and UV-blocking performance, the SPI–CNF–NA composite film prepared also exhibits strong inhibition towards *A. niger*, one of the main fungal species responsible for blueberry spoilage.

### 3.3. Potential of SPI–CNF–NA Composite Film in Blueberry Preservation

#### 3.3.1. Weight Loss Ratio of Blueberries

The weight loss rates of fresh blueberries stored at 4 °C for 20 days are illustrated in Figure 6e. The blueberries from the four treatment groups all lost weight during the early storage stage. However, their weight loss rates differed significantly at the end of the storage period, depending entirely on the types of film tested. Compared to the control group without film treatment, the weight loss rate of blueberries treated with three SPI-based films slowed down after 10 days of storage. The weight loss rate of blueberries was 16.32% for the control group, 14.61% for the SPI film group, 13.86% for the SPI–CNF film group, and 11.70% for the SPI–CNF–NA composite film group after 20 days of storage. It was clear that the SPI–CNF–NA composite film exhibited the best ability in reducing the weight loss of fresh blueberries during a 20-day shelf life.

#### 3.3.2. Decay Rate of Blueberries

The decay rates of blueberries under the four treatments are shown in Figure 6f. The decay rate of the blueberries accelerated starting from the 15th day of storage but depended largely on the film types tested. The decay rate of blueberries was 22.66% for the control group, 18.67% for the SPI film group, 14.0% for the SPI–CNF film group, and 11.0% for the SPI–CNF–NA composite film group. Of all the treatments, the decay rate of blueberries treated with SPI–CNF–NA composite film was the lowest at the end of the storage period, at 39.02% lower than that of the control group. Our results showed that using SPI–CNF–NA composite film to treat blueberries effectively reduced the spoilage of the fruit.

#### 3.3.3. Appearance and Firmness Performance of Blueberries

**Figure 7:**
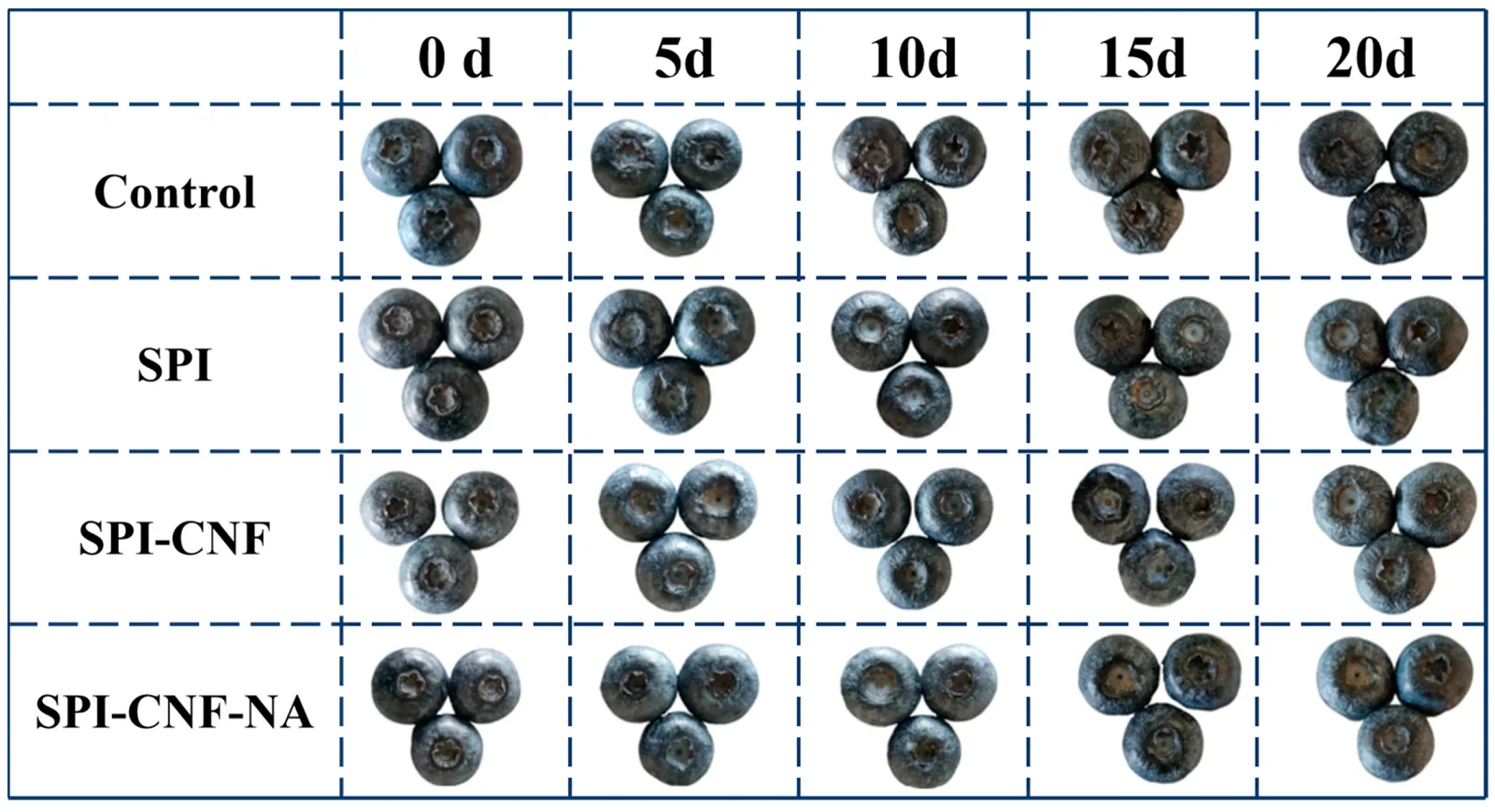
Visual appearance of blueberries unwrapped and covered with different films and stored from day 0 to day 20.

**Figure 8:**
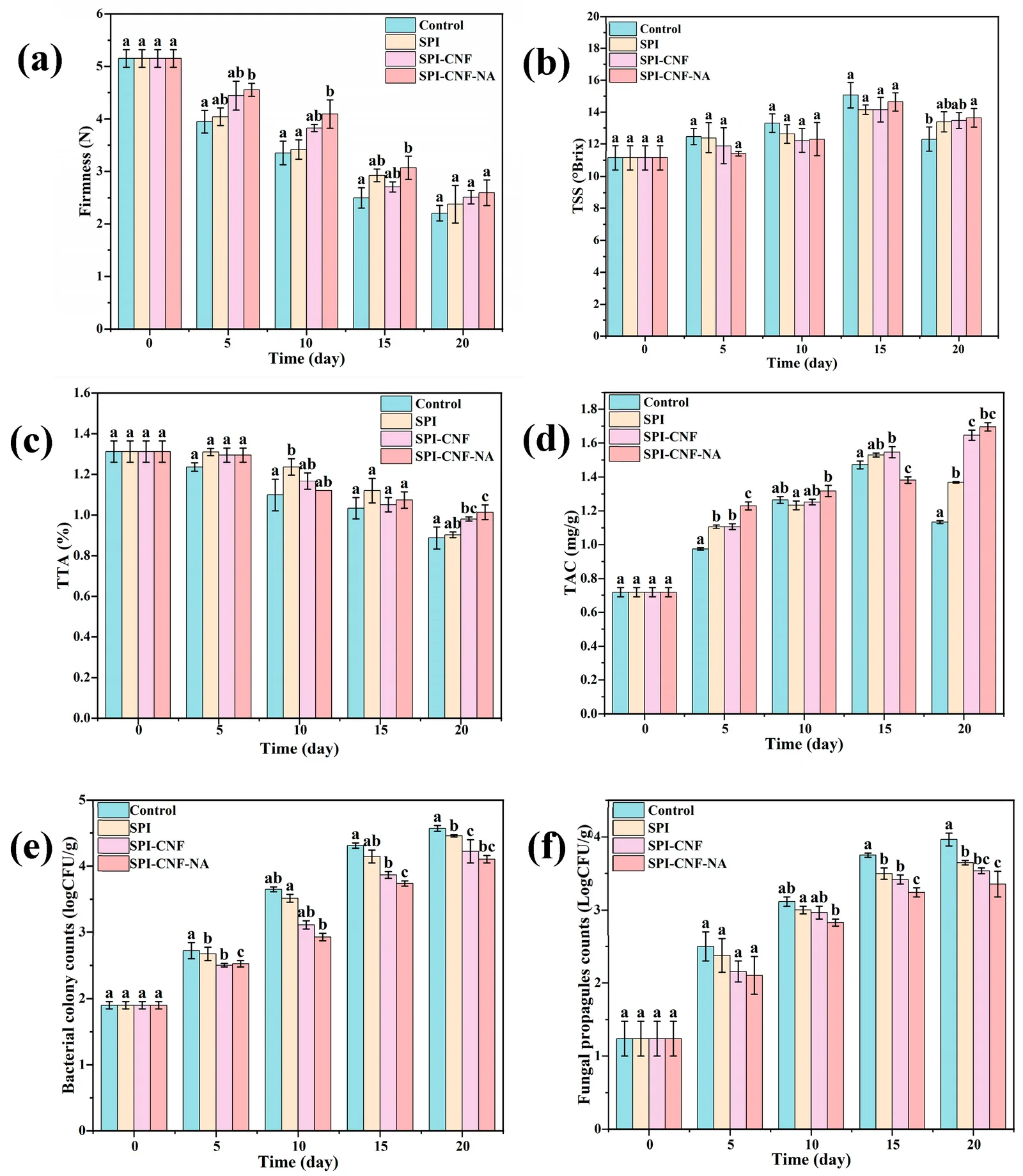
Changes in the physicochemical parameters of postharvest blueberries covered with different films during a 20-day storage period. (**a**) Firmness, (**b**) total soluble solids (TSS), (**c**) total titratable acidity (TTA), (**d**) total anthocyanin content (TAC), (**e**) total bacterial colony counts, and (**f**) total fungal propagules counts of blueberries sampled during the storage period. Data are expressed as mean ± SD (n = 3). Different lowercase letters denote significant differences among groups at *p* < 0.05, as determined by one-way ANOVA followed by Tukey’s multiple range test.

The impacts of different composite films on the appearance and firmness of blueberries are depicted in Figure 7 and Figure 8a, respectively. The firmness of blueberries consistently lowered but depended on the treatments throughout the storage period. During the early storage stage, the firmness of blueberries declined rapidly in the control and SPI groups, whereas the firmness of blueberries treated with SPI–CNF and SPI–CNF–NA composite films slowed down after experiencing a relatively rapid decrease on the 15th day. The firmness values of blueberries were 2.20 N for the control group, 2.37 N for the SPI film group, 2.50 N for the SPI–CNF film group, and 2.59 N for the SPI–CNF–NA composite film group after 20 days of storage. The appearance of fresh blueberries sampled for this study was uniform, characterized by a blue color, plump shape, glossy surface, firm texture and distinct aroma (Figure 8a). During a 20-day storage period, the blueberries gradually softened, developed wrinkled surfaces, lost moisture and showed tissue damage. However, the degree of appearance changes in the blueberries largely depended on the SPI-based film treatments. The blueberries treated without film as the control developed a deep blue hue with severe wrinkling and mild shrinkage at the endpoint of the storage period. The blueberry fruits treated with the SPI–CNF–NA composite film retained a much better exterior appearance, with less wrinkling, an intact fruit shape, and negligible color discoloration (Figure 8a). Obviously, the use of SPI–CNF–NA composite film to treat blueberries could preserve the original hardness and appearance of the fruit better.

#### 3.3.4. Total Soluble Solid Content of Blueberries

For the four treatments, the TSS content of blueberries exhibited a consistent trend that first increased and then decreased throughout the storage period (Figure 8b). The maximal TSS levels of blueberries from the control (15.08 °Brix) and SPI groups (14.16 °Brix) appeared on the 10th day. However, the maximal TSS levels of the blueberries treated with the SPI–CNF (14.16 °Brix) and SPI–CNF–NA (14.66 °Brix) films appeared on day 15. It was noted that the blueberry fruits treated with the SPI–CNF–NA composite film maintained higher TSS contents at the endpoint of storage period than the other three treatments. Clearly, the SPI–CNF–NA composite film may therefore be helpful for maintaining the TSS content of blueberries.

#### 3.3.5. Total Titratable Acidity of Blueberries

The TTA values of blueberries from the four treatments are illustrated in Figure 8c. The TTA level of blueberries exhibited a continuous downward trend as the storage time was delayed to 20 days. After day 10, the TTA values of the blueberries declined notably more rapidly, although this differed among the treatments. The TTA value of blueberries reduced by 32.39% for the control group from day 0 to the 20th day of storage, whereas it decreased by 25.30% for the SPI–CNF group and by 22.78% for the SPI–CNF–NA group.

#### 3.3.6. Total Anthocyanin Content of Blueberries

The TAC values of blueberries under the four treatments during a 20-day storage period are exhibited in Figure 8d. The TAC of blueberries increased over the 20 days, but this differed among the treatments. The TAC content of blueberries sampled from the control group reached a maximum but then quickly decreased to 1.13 mg/g on day 20, whereas that of blueberries treated with the SPI–CNF and SPI–CNF–NA composite films showed a continuous increase throughout the storage period. The TAC of blueberries was 1.36 mg/g for the SPI group, 1.64 mg/g for the SPI–CNF group, and 1.69 mg/g for the SPI–CNF–NA group after 20 days of storage. Clearly, the SPI–CNF–NA composite film preserved blueberry anthocyanin more effectively.

#### 3.3.7. Microbiological Analysis of Blueberries

The microorganism numerations of blueberries sampled from the four treatments during a 20-day storage period are presented in Figure 8e,f. The bacterial colony counts of the blueberries averaged 79.43 CFU/g, and the viable fungal propagules counts averaged 17.38 CFU/g on day 0. Despite exhibiting a consistent increase across the storage time, the bacterial and fungal viable counts of blueberries differed entirely among the treatments at the end of the storage period. The live bacterial counts of the blueberries were 3.73 × 10^4^ CFU/g for the control group, 2.88 × 10^4^ CFU/g for the SPI group, 1.68 × 10^4^ CFU/g for the SPI–CNF group, and 1.28 × 10^3^ CFU/g for the SPI–CNF–NA group after 20 days. The viable fungal propagule counts of the blueberries were 9.12 × 10^3^ CFU/g for the control group, 4.42 × 10^3^ CFU/g for the SPI group, 3.42 × 10^3^ CFU/g for the SPI–CNF group, and 2.23 × 10^3^ CFU/g for the SPI–CNF–NA group. Clearly, compared to the other three treatments, SPI–CNF–NA composite film is most effective at reducing the bacterial and fungal counts of blueberries during a 20-day storage period. Based on the above research, it can be concluded that the SPI–CNF–NA composite film extended the shelf life of blueberries by approximately 5 days compared with the control group.

## 4. Discussions

### 4.1. High-Quality CNF Should Be Available for Preparing SPI-Based Composite Film

The CNF obtained from soybean hulls was of the high quality required for fabricating SPI-based composite films, as evidenced by its ultrafine particle size (≈9.71 nm) and clean surface free of visible impurities. This nanocellulose morphology directly contributed to the enhanced diffraction peak intensities observed in our XRD patterns (Figure 2b), which are closely associated with the uniform and ultrafine particle size of CNF. This is consistent with the well-documented role of high-quality CNF as a functional filler in food preservation films [29,30]. CNF enhanced the diffraction peak intensities, which are closely associated with its ultrafine, uniform particle size. Specifically, the increased intensity of the absorption peak at 3418 cm^−1^ in CNF is attributed to abundant hydroxyl groups and strengthened intermolecular hydrogen bonds generated through DES treatment [31]. Comparable nanofiller quality (with CNF diameters of 3–50 nm) has likewise been shown to confer excellent mechanical performance, high thermal stability, and superior biocompatibility to polymer composites [32]. In our case, the CNF prepared maintained the inherent cellulose I crystal structure, indicating that the current chemical treatments did not alter the cellulose structure, which is critical for preserving the structural integrity of cellulose-based composites and thus maintaining good thermal stability [33]. DES-modified CNF derived from soybean residue presents higher thermal stability than raw cellulose, which can be attributed to the larger specific surface area and enriched surface hydroxyl groups of CNFs after DES modification [34]. Moreover, our results showed that nano-crystallization-induced structural changes and crystallinity variations significantly improved the physicochemical properties of cellulose-based materials, including excellent barrier performance and good mechanical properties, making it an ideal filler for the preparation of SPI–CNF composite films (see Figure 2). The methodological result is that CNF with superior film-forming properties shows great promise for high-performance food-contact preservation applications [35]. Additionally, our study verified that using alkaline and bleaching to remove impurities such as hemicellulose and lignin from soybean hulls, followed by DES to treat the cellulose, provides a promising method for extracting high-purity nanoscale cellulose, i.e., CNF.

### 4.2. Optimal Amounts of CNF and NA Guarantee the Functionality of the SPI–CNF–NA Composite Film

In our study, it was discovered that the overall performance of SPI-based composite films was highly dependent on the filler dosage. Low-dose CNF (1.0 to 3.0%) was observed to effectively reduce the WVTR of the SPI–CNF film. This improvement stems from hydrogen bonding between hydroxyl groups on CNF surfaces and amino/carboxyl groups of SPI or hydroxyl groups of glycerol. Such intermolecular interactions weaken the matrix hydrophilicity and improve the water retention capacity of composite films, thereby blocking the penetration and diffusion of water vapor molecules inside the film. In turn, excessive CNF addition increases film hydrophilicity and thus transfers more moisture from inside to outside the film due to the exposure of abundant surface hydroxyl groups [36]. This behavior is closely correlated with film homogeneity and the size and quantity of internal void defects. As a high-aspect-ratio one-dimensional nanofiller, the reduced OP of SPI–CNF composite film is attributed to the intermolecular entanglement induced by CNF within the film matrix, which forms a tortuous pathway to hinder oxygen diffusion. Additionally, CNF serves as a rigid reinforcing filler that compacts the film microstructure and reduces internal porosity, further improving oxygen barrier performance [37]. The improved TS is ascribed to the high aspect ratio of CNF, which strengthens interfacial interactions between CNF and the SPI matrix. Enhanced interfacial adhesion improves the internal cohesion of film components, thereby increasing the fracture resistance of composite films. Of course, the decreased EAB results from the restricted mobility and conformational flexibility of SPI molecular chains caused by strong SPI–CNF interactions. Our findings are consistent with the previously reported results of SPI/cotton-derived CNF composite films [38]. In our case, a 3.0% CNF was identified as the optimal addition dosage for maintaining the excellent properties of the fabricated SPI–CNF composite film.

Moreover, we found that the addition of NA (1.0 to 5.0%) gradually reduced the WVTR of the SPI–CNF–NA composite film due to the enhanced intermolecular interactions among the components of the film and the optimized pore structure with reduced pore size. Our study found that high NA dosages (4.0 to 5.0%) slightly increased the OP of the SPI–CNF–NA composite film. This phenomenon originates from the poor solubility of NA in the film-forming matrix, which triggers spontaneous NA aggregation and localized structural defects. The increased regional porosity destroys the integrity of the oxygen barrier network and weakens the overall barrier performance [39]. Meanwhile, progressive increases in NA dosage (1.0 to 5.0%) led to simultaneous declines in TS and EAB. The inferior mechanical performance is primarily caused by the poor aqueous solubility of NA, which produces mechanically weak domains inside the SPI–CNF–NA composite film. These heterogeneous structural defects act as stress-concentration sites under external loading, accelerating crack initiation and propagation, and thereby resulting in premature fracture and deteriorated ductility of the composite film [40]. Meanwhile, excessive NA addition reduced its encapsulation efficiency due to poor solubility [41]. On the other hand, the addition of NA gave the SPI–CNF–NA composite film good antifungal activity against the growth of *A. niger*, a humidity-dependent fungus that thrives in high-moisture conditions. Evidently, the optimal amount of NA added should be selected to improve not only the antifungal action against *A. niger* of the SPI–CNF–NA composite film but also its barrier performances. In our case, 3.0% NA was identified as the optimal amount to add to maintain an excellent water vapor and oxygen barrier and the mechanical and antifungal properties of the fabricated SPI–CNF–NA composite film. The optimized SPI–CNF–NA composite film was fabricated by incorporating CNF (3.0%) and NA (3.0%) into a matrix of SPI plus glycerol.

### 4.3. How SPI/CNF/NA Contribute to the Potential of the Composite Film

Achieving a clear understanding of the SPI–CNF–NA composite film is vital for preserving blueberries. First, high transparency ensures that the composite film meets the basic visual requirements for blueberry packaging, although the addition of NA leads to slight roughness and dispersed small particles [42]. Thus, we selected 3.0% NA and 3.0% CNF as the optimal amounts to add to preserve the smooth, homogeneous surfaces of the SPI–CNF–NA composite film and maintain the composite film’s high transparency. Our results showed that, compared to the SPI film, the absence of new absorption peaks in the SPI–CNF and SPI–CNF–NA composite films confirmed the compatibility among SPI, CNF and NA, which lay the structural foundation for stable film formation and functional integration. The blue shift of the peak from 3276 to 3280 cm^−1^ and 3282 cm^−1^, the decreased intensities of the amide I, II, and III bands, and the increased intensity of the peak at 1038 cm^−1^ demonstrated that the formation of hydrogen bonds between CNF and glycerol replaced native protein–protein interactions in the SPI matrix and gave rise to new CNF–protein and NA–protein intermolecular interactions [43].

Thus, improved plasticizer retention and film homogeneity should be attributed to the involvement of CNF and NA, which disrupts the intrinsic hydrogen-bonding network of the SPI matrix, as indicated by our FITR and SEM data. Hydrogen bonds were formed among the functional groups of the SPI matrix, glycerol, and the hydroxyl groups of licorice residue nanocellulose [44]. Similarly, previous studies have reported that incorporating less than 7% pea shell-derived cellulose nanocrystals into carboxymethyl cellulose composite films preserved surface smoothness [45]. Inherently, the SPI matrix is well-known to exhibit UV-shielding activity. Our study found that the incorporation of CNF strengthened the ultraviolet barrier capacity of the SPI–CNF composite film. Subsequent addition of NA further enhanced the SPI–CNF–NA composite film’s ability to block UV due to an effect that is possibly derived from the hydrophobic moieties of NA [46]. The tolerance of the SPI–CNF and SPI–CNF–NA composite films to heat, compared to the single SPI film, is reflected by their distinct thermal decomposition stages. The regulatory effect of CNF and NA on the thermal stability of the composite film matrix is attributed to the replacement of the intrinsic decomposition pathway of SPI by glycerol–polymer sequential decomposition, which is advantageous for controlled thermal stability in packaging applications. The three films exhibited satisfactory thermal tolerance, and the addition of CNF and NA slightly enhanced their resistance towards thermal decomposition. Our results are consistent with previous work demonstrating that the incorporation of cellulose into pectin/carrageenan films yields comparable thermal degradation profiles for cellulose-based materials [47]. Furthermore, the enhanced thermal decomposition tolerance of SPI-based composite films confirms their suitability for food packaging applications under mild thermal processing or ambient temperature-fluctuation conditions [48]. The addition of CNF and NA reinforced the preservation of the SPI–CNF–NA composite film.

### 4.4. The Potential of the SPI–CNF–NA Composite Film for Preserving Blueberries

In our case, blueberries suffered multiple deteriorative changes in external quality after a 20-day storage period without SPI–CNF–NA composite film treatment. Defects in the appearance of the blueberries included water loss-induced shrinkage, weight decline, and skin oxidative discoloration. Rapid water transpiration dominated weight loss at the early storage phase, followed by respiratory consumption of organic substrates. The fruit firmness of the blueberries without the SPI–CNF–NA composite film treatment decreased due to the destruction of cell wall mechanical strength, pectin solubilization and intercellular adhesion [49,50]. However, the SPI–CNF–NA composite film formed a dense protective barrier layer on the blueberry surface, effectively limiting the moisture and oxygen migration, slowing physiological metabolism, and preventing pectin and starch hydrolysis, thereby retarding ripening and cellular senescence [51]. Furthermore, the restricted oxygen infiltration effectively alleviated excessive phenolic oxidation and melanin accumulation on the fruit peel, thereby mitigating surface wrinkling, shriveling and browning symptoms. The blueberries treated with the SPI–CNF–NA composite film exhibited the best appearance, which greatly improved the fruit’s consumer appeal and commercial value after a 20-day storage period. The SPI–CNF–NA composite film minimized weight loss, maintained the cell-wall structural integrity and fruit firmness, and alleviated shrinkage and wrinkling and excessive pigment oxidation by virtue of its excellent water barrier capacity. A comparison between the control and the SPI–CNF–NA composite film demonstrated the essential role of the composite film in maintaining the appearance of post-harvested blueberries [52].

Generally, total soluble solids (TSS), total titrating acids (TTA), and total anthocyanins content (TAC) are used to describe the loss of internal nutritional quality of blueberries stored for a regular period [53]. In this study, the increase in TSS during the early storage stage of blueberries was attributed to multiple physiological events, including organic acid decomposition, starch-to-sugar conversion, hydrolysis of cell-wall polysaccharides, and sugar concentration induced by water loss. A reduction in TSS, on the other hand, should depend on continuous respiratory consumption, microbial spoilage, and extensive nutrient degradation in the late storage period [54]. Compared with the other treatments, the TSS of blueberries treated with the SPI–CNF–NA composite film increased more slowly, due to oxygen permeation being blocked. This indicates that using the SPI–CNF–NA composite film could prevent blueberry nutrients from degrading rapidly by decreasing respiration, as seen in the firmness changes [55]. The progressive reduction in TTA across all treatments was directly related to the inherent postharvest physiological metabolism of blueberries [56]. However, the TTA of blueberries treated with the SPI–CNF–NA composite film treatment only decreased by 22.78% compared to the initial level. This suggests that, compared to other treatments, the SPI–CNF–NA composite film may block the conversion of organic acids from blueberries to soluble sugars more effectively. It was observed that the TAC of blueberries increased during the early storage stage but declined in the late storage stage, largely depending on the treated groups. A significant decline in the TAC level of blueberries was observed in the control and SPI groups compared to the SPI–CNF–NA treatment. Obviously, the SPI–CNF–NA composite film effectively prevented the accumulated anthocyanins from degrading rapidly by decreasing the oxidation and decomposition of the phenolic compounds. This phenomenon may be attributed to the accelerated synthesis and accumulation of phenolic compounds and their glycosidic conjugations in blueberries that outpace their degradation rate during long-term postharvest storage and fruit senescence [57].

The SPI–CNF–NA composite film significantly reduced the decay rate of blueberries by inhibiting the growth of microorganisms due to the presence of NA and the film’s barrier against microbial infections. In our case, moderate decay of blueberries took place during their early storage stage, yet decay accelerated after 15 days due to a typical postharvest physiological phenomenon driven by microbial proliferation [58], largely depending on the treatments. The bacterial colony counts and fungal propagules counts of blueberries treated with the SPI–CNF–NA composite film were found to be significantly lower than for other treatments. The superior performance of the SPI–CNF–NA composite film in preserving blueberries from quick spoilage should be primarily attributed to the inherent antifungal activity of NA, complemented by the barrier property of the film, which restricts microbial invasion. As previously documented, NA-loaded composite films mitigate natural-infection-caused tomato decay while preserving fruit quality; meanwhile sodium-alginate-NA films maintain cell-wall integrity and delay fruit softening and peel browning [59]. We applied the SPI–CNF–NA composite film to enhance the resistance of postharvest blueberries to spoiled microorganisms during a 20-day storage period, thereby reducing not only the total bacterial colony counts and fungal propagules counts but also the decay rate of the fruit [60]. In postharvest preservation studies of blueberries, total bacterial colony counts above 10^4^ CFU/g and fungal propagule counts exceeding 10^3^ CFU/g are generally regarded as indicative of unacceptable fruit quality. In this study, the control group reached these microbial thresholds at approximately day 15 of storage, whereas the SPI–CNF–NA-treated blueberries delayed this limit being reached by approximately 5 days, which aligns with the observed appearance and firmness evolution of the fruits.

Although sensory evaluations of taste and fruit aroma were omitted in this study, its novelty lies in the development of an SPI–CNF–NA active composite film. Unlike single-component protein films, the incorporation of CNF and NA simultaneously improves the mechanical and oxygen barrier performance of the SPI matrix, while endowing the film with antifungal activity against *A*. *niger*. This study further validated that the composite packaging effectively delayed weight loss and decay incidence of blueberries. As such, the fabricated SPI–CNF–NA composite film is a promising tool for maintaining the original appearance characteristics and nutritional quality of postharvest blueberries by slowing down physiological ripening and reducing decay caused by the fruit being attacked by spoiled microorganisms.

## 5. Conclusions

Preparing high-quality CNF extracted from soybean hull is a prerequisite for successfully manufacturing SPI-based composite films for preserving postharvest blueberries. Subsequently, the optimized SPI–CNF–NA composite film was fabricated by incorporating CNF (3.0%) and NA (3.0%) into a matrix of SPI plus glycerol. The incorporation of CNF and NA not only enhanced the barrier activity of the SPI–CNF–NA composite film against water vapor, oxygen and UV radiation, but also strengthened the film’s antifungal activity against *A*. *niger* CICC 40868; its inhibition zone diameter reached 35.22 mm. Moreover, the addition of CNF and NA did not compromise the thermal decomposition temperature of the SPI–CNF–NA composite film due to the synergistic effect of the SPI/CNF/NA components, which collectively reinforced the fabricated film’s ability to preserve freshness. Importantly, compared with the control group, using the SPI–CNF–NA composite film reduced the weight loss from 16.32% to 11.70% and the decay rate from 22.66% to 11.0%. Meanwhile, the film maintained firmness and nutritional quality by improving barrier properties, lowering microbial proliferation, and effectively delaying the ripening and senescence of the fruits. Finally, the shelf-life of the SPI–CNF–NA-treated blueberries was extended by approximately 5 days compared with the control group. The limitations of this study include the absence of sensory evaluation and antimicrobial testing being limited to a single fungal strain. Future work will focus on incorporating sensory evaluation and consumer acceptance tests, expanding antimicrobial testing to a wider range of fungi, bacteria and yeasts, and validating the film performance on different horticultural crops under commercial storage conditions. To our knowledge, this study should provide the first report that provides a promising technology for maintaining the freshness of postharvest blueberries.

## Figures and Tables

**Table 1:** The diameter of the inhibition zone of NA casting solution with different contents.

The Amount of NA Added (%)	Inhibition Zone Diameter (mm)
0	0
1	27.52 ± 0.81 ^a^
2	30.70 ± 0.16 ^b^
3	34.09 ± 0.76 ^d^
4	35.22 ± 0.03 ^e^
5	32.82 ± 0.13 ^c^

Data are expressed as mean ± standard deviation. Different superscript letters in the same column indicate significant differences (*p* < 0.05) according to Tukey’s test.

## Data Availability

The original contributions presented in this study are included in the article. Further inquiries can be directed to the corresponding authors.
